# Antimicrobial peptide DiPGLa-H exhibits the most outstanding anti-infective activity among the PGLa variants based on a systematic comparison

**DOI:** 10.1128/aem.02062-24

**Published:** 2025-02-05

**Authors:** Liangjun Zheng, Muhammad Zafir, Ziqian Zhang, Yadong Ma, Fengyi Yang, Xiaokun Wang, Xuemei Xue, Chen Wang, Ping Li, Pilong Liu, Fatma A. El-Gohary, Xin Zhao, Huping Xue

**Affiliations:** 1 Department of Animal Science and Technology, University of Northwest A&F 12469 , Yangling, Shaanxi, China; 2 Olymbel Bioengineering Institute, Zhangye, Gansu, China; 3 Department of Hygiene and Zoonoses, Faculty of Veterinary Medicine, Mansoura University 158400 , Mansoura, Egypt; 4 Department of Animal Science, McGill University 317104 , Sainte-Anne-de-Bellevue, Québec, Canada; Centers for Disease Control and Prevention, Atlanta, Georgia, USA

**Keywords:** AMPs, DiPGLa-H, DAMP4, biosynthesis, non-chromatographic purification

## Abstract

The escalating threat of antibiotic-resistant bacteria has heightened global interest in antimicrobial peptides as promising candidates due to their potent broad-spectrum activity and low likelihood of resistance development. Despite this potential, these peptides face challenges, including modest bactericidal efficacy, insufficient safety assessment, and expensive production. In this study, we systematically evaluated a panel of nine AMP variants of PGLa, a natural AMP derived from *Xenopus laevis*. All peptides retained α-helical structures and exhibited high biocompatibility, with hemolytic concentrations above 128 µg/mL and macrophage survival rates over 80%. Among them, a tandem-repeat variant DiPGLa-H demonstrated the most potent antimicrobial activity, with a therapeutic index of 35.94, against key pathogens such as *Escherichia coli, Staphylococcus aureus,* and *Acinetobacter baumannii*. A DAMP4-DiPGLa-H fusion protein was engineered to mitigate potential host toxicity, and we achieved high-purity biosynthesis of DiPGLa-H by employing a combination of acid cleavage and non-chromatographic purification, with yields reaching 21.2 mg/mL. The biosynthesized DiPGLa-H exhibited robust stability across a wide pH range and high temperatures, effectively disrupting biofilms formed by multiple pathogenic species. Mechanistically, DiPGLa-H disrupts both the inner and outer bacterial membranes, causing cell shrinkage, vesiculation, and intracellular leakage. *In vivo*, DiPGLa-H significantly improved survival rates in mice with induced peritoneal inflammation by 31%–38% while reducing bacterial burdens in key organs by 100-fold to 1,000-fold. These findings unearthed DiPGLa-H as a highly promising AMP. Moreover, the successful development of a cost-effective, high-purity biosynthesis method for DiPGLa-H, utilizing DAMP4 fusion technology, enables its low-cost application in combating multidrug-resistant pathogens.

**IMPORTANCE:**

AMPs are innate defense molecules in animals, plants, and microorganisms. Notably, one-third of these peptides in databases originate from amphibians. We discovered that naturally weak AMPs from this source can be enhanced through artificial design. Specifically, variant DiPGLa-H showed superior germicidal efficacy and cell selectivity both *in vivo* and *in vitro* and can be biosynthesized and purified by combining DAMP4 fusion protein strategy and a simple non-chromatographic method that facilitates large-scale production. Our focus is on understanding the structure-activity relationships of PGLa. Furthermore, the development of a non-chromatographic purification technique for AMPs offers a viable pathway for the large-scale production of these essential compounds.

## INTRODUCTION

Antimicrobial peptides (AMPs) encompass a diverse group of molecules produced by both prokaryotic and eukaryotic organisms. They exhibit broad-spectrum antimicrobial activity, rapid bactericidal action, and a lower propensity for resistance development, primarily due to their multifaceted modes of action and ability to target multiple microbial components (1). It is important to note that AMPs are not universally low-molecular-weight peptides, as their lengths can vary widely, often ranging from 10 to over 100 amino acids. Despite these variations, AMPs are considered promising candidates for the development of next-generation therapeutic agents against multidrug-resistant pathogens (2). The Antimicrobial Peptide Database (APD3) (https://aps.unmc.edu/) catalogs 3,569 AMPs culled from diverse sources including humans, animals, plants, and microorganisms (3). Among these peptides, 1,196 are of amphibian origin, primarily due to the rich reservoir of biologically active peptides within amphibian skin, facilitating their facile discovery, purification, and characterization (4). These amphibian skin-derived peptides hold immense promise for therapeutic applications in diverse fields, encompassing cancer, human immunodeficiency virus (HIV), and the combat of methicillin-resistant *Staphylococcus aureus* (MRSA) (5). Therefore, the structural and functional exploration of active peptides within amphibian skin secretions is deemed imperative for the advancement of innovative antimicrobial agents.

PGLa, an amphibian 21-residue cationic AMP, with a net charge of +4, was found in the skin of the toad *Xenopus laevis* (6). The minimum inhibitory concentration (MIC) of PGLa against *Escherichia coli* 25922 and *S. aureus* 25923 were 10–50 μg/mL and 50–100 μg/mL, respectively (6). PGLa variant PGLa-H isolated from the skin of African clawed frog had a MIC value of 8.7 µg/mL against *S. aureus* ATCC 25923 and 23.6 µg/mL against *E. coli* ATCC 25922 (7). Although DiPGLa-H was generated by tandem repetition of PGLa-H, exhibiting MIC values of 0.75–1.5 μM against *E. coli* and *S. aureus* (8). The replacement of Val-Gly within kiadin-1 at specific positions resulted in decreased hemolytic activity while maintaining antimicrobial efficacy, likely due to enhanced flexibility in its helical structure (8). Subsequent glycine substitution mutations in kiadin-1 resulted in the derived peptide, kiadin-2, which exhibited enhanced bactericidal activity against Gram-negative pathogens. In addition, other natural variants of PGLa include PGLa-B1, PGLa-B2, and PGLa-AM1. These variants exhibited MIC values of 12.5 µM, 25 µM, and 12.5 µM against *E. coli*, and 25 µM, 50 µM, and 25 µM against *S. aureus*, respectively (9). The natural variant PGLa-MW1, identified from the skin secretions of *X. muelleri*, demonstrated a MIC of 12 µM against *E. coli* and a weak inhibitory activity against *S. aureus* (MIC = 100 µM) (10).

To date, there is a lack of systematic comparative study on the bacteriostatic activity and biosafety of PGLa and its variants, which precludes the determination of the variant with the highest potential for application. It has been more than 30 years since the discovery of PGLa in 1983 to the recent discovery of new PGLa-derived peptides. During this long research period, due to the change of theory and technology, the results of different studies are quite different and not comparable, and there is a lack of clear animal test results and clear bacteriostatic mechanism; hence, it is impossible to determine which peptide in PGLa and its variants has the most outstanding application value. For example, the MIC of kiadin-1 to *Pseudomonas aeruginosa* ATCC 25922 are 6 µM and >32 µg/mL, and the MIC to MRSA were 3 µM and 16 µg/mL, respectively, in two independent studies (11, 12). The MIC of PGLa-AM1 against *Helicobacter pylori* and *Acinetobacter nosocomial* Ab113 were 1 µg/mL and 128 µg/mL, respectively (13, 14). Intriguingly, a systematic exploration of the antimicrobial attributes of PGLa and its derivative variants has been notably absent from the scientific discourse.

In this study, we synthesized a total of nine peptides via solid-phase method, encompassing PGLa and its variants. Subsequently, we conducted an in-depth analysis of their physicochemical characteristics and helical wheel projections, as well as their secondary structures both in aqueous conditions and within membrane-mimicking environments. Peptide DiPGLa-H was identified as the most valuable one among PGLa variants, due to its superior cellular selectivity, remarkable *in vitro* stability, and resistance to biofilm. Additionally, a high concentration and high purity of DiPGLa-H was produced biologically, by combining with DAMP4-DiPGLa-H fusion protein strategy and non-chromatographic purification. The biosynthesized DiPGLa-H was subjected to rigorous assessment under a spectrum of *in vitro* conditions, and the results indicated that DiPGLa-H exhibited robust stability across a wide pH range and high temperatures. Further investigations aimed at unraveling the bactericidal mechanisms exhibited by these peptides were initiated, by employing an array of techniques as fluorescence probes, microscopy modalities, and electron microscopy. The results showed DiPGLa-H disrupted bacterial membranes and caused cell shrinkage, vesiculation, and intracellular leakage. Finally, an anti-infection experiment showed DiPGLa-H significantly improved survival rates in mice with induced peritoneal inflammation while reducing bacterial burdens in key organs.

## RESULTS

### Nine variants of PGLa exhibit consistent α-helical structure but differentiated physicochemical properties

**Fig 1:**
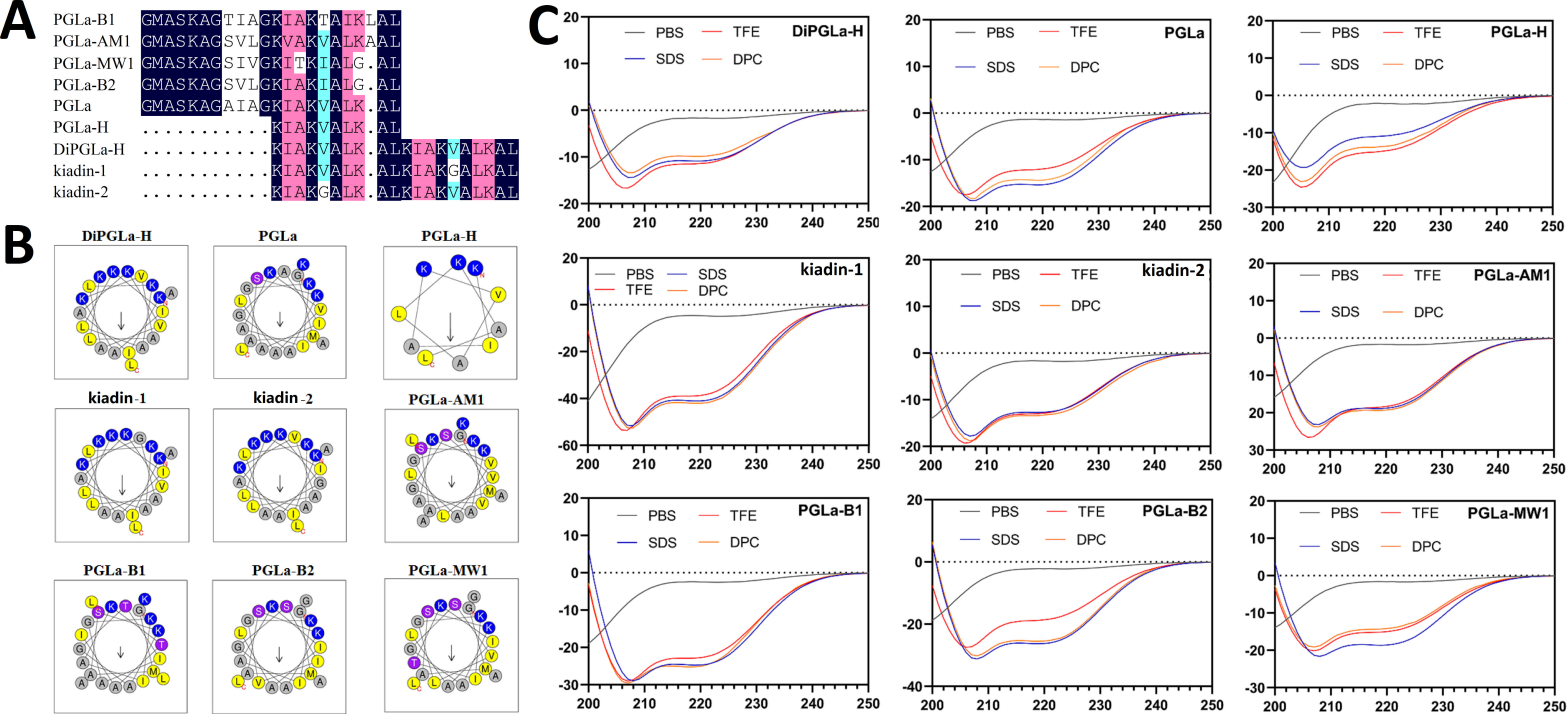
Structure and physicochemical properties of nine PGLa variants. (**A**) Comparison of amino acid sequences of AMPs PGLa and variants. Those with sequence identity of 100%, 75%–99%, and 50%–74% are marked as dark blue, pink, and light green, respectively. (**B**) Helical wheel projections of peptides. Among these helical wheel projections, by default the output presents the charged residues as blue, hydrophobicity residues as yellow, uncharged residues as light pink, and alanine and proline as gray and green, respectively. The longer the arrow length, the greater the relative hydrophobic moments in the figure. (**C**) CD spectra of engineered peptides. Engineered peptides were dissolved in 10 mM sodium phosphate buffer (pH 7.4) (blue), 30 mM SDS (green), 50% TFE (red), and 10 mM DPC (orange). The mean residue ellipticity was plotted against wavelength. The values from three scans were averaged per sample, and the peptide concentrations were fixed at 150 mM.

To elucidate the functional distinctions among various PGLa-derived peptides, nine derivative variants were chemically synthesized and thoroughly characterized (Fig. 1A). Comparative analysis revealed congruence between the theoretical and observed molecular weights for these peptides (Table S1). A common consensus attributes the antibacterial efficacy of AMPs to their hydrophobicity. Accordingly, we determined and ranked their hydrophobicity as follows: PGLa-B2 >PGLa-MW1 >DiPGLa-H=PGLa H >PGLa-B1 >kiadin-1=kiadin-2 >PGLa > PGLa-AM1. It is noteworthy that PGLa-B2, PGLa-MW1, DiPGLa-H, and PGLa-H exhibited hydrophobicity values exceeding 0.4 (average: 0.449). A critical factor in the initial interaction of cationic AMPs with negatively charged cell membranes is the positive charge they carry. DiPGLa-H, kiadin-2, and kiadin-1 bore the highest positive charge, each possessing six charges, whereas other peptides featured 3–4 charges. Further peptide helical wheel projections (Fig. 1B) and relative hydrophobic moments (Table S1) indicated that PGLa-H displayed ideal amphipathic properties (μHrel = 0.551), exemplifying an even alignment of its hydrophobic and cationic faces.

To gain insight into the conformational dynamics of peptides under varying environmental conditions, circular dichroism analyses were conducted on nine PGLa-derived peptides in PBS, SDS, dodecylphosphocholine (DPC), and trifluoroethanol (TFE) solutions (Fig. 1C). TFE, functioning as a helix-inducing agent, simulated the hydrophobic surroundings akin to bacterial cell membranes, enabling the assessment of the helical propensity of peptides. In contrast, DPC micelles was utilized to mimic the lipid bilayers environment, which can more accurately reflect the structural transformation of peptides in the lipid bilayers environment. Negatively charged SDS micelles emulated anionic membrane environments. The results disclosed that none of the peptides exhibited discernible α-helical structural features in PBS, displaying an obviously local minimum dichroic band at approximately 200 nm (Fig. 1C). Conversely, when exposed to 10 mM DPC, 50% TFE solution, and 30 mM SDS micelles, all peptides displayed two minimum dichroic bands at approximately 208 nm and 222 nm, a characteristic signature of an α-helical structural propensity (Fig. 1C).

### DiPGLa-H exhibits the strongest bacteriostatic and clinical applicability among nine variants

**Fig 2:**
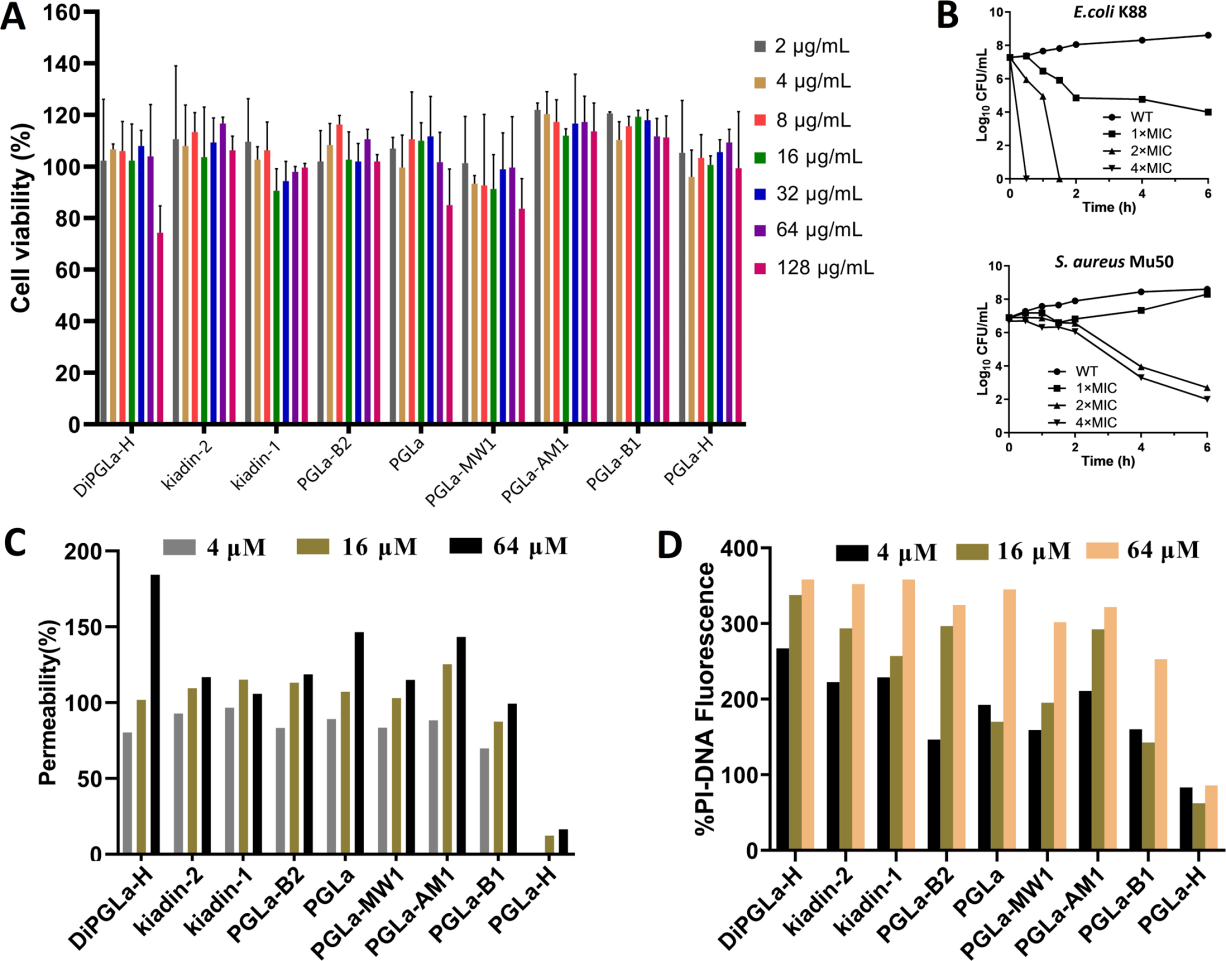
Assessment of peptide cytotoxicity, antibacterial effects, and membrane interaction. (**A**) Cytotoxicity of the engineered peptides against RAW 264.7 cells. (**B**) Bactericidal ability of DiPGLa-H against *E. coli* and *S. aureus*. (**C**) Determination of the permeability of nine AMPs to the outer membrane of *E. coli* by NPN. (**D**) Determination of permeability of nine AMPs to *E. coli* intima by PI test.

Hemolytic activity and cytotoxicity assessments are pivotal for gauging the safety of antimicrobial agents. We initiated our evaluation by determining the minimum hemolytic concentration (MHC) for all peptides, except PGLa-B1, which induced hemolysis exceeding 10% in pig erythrocytes at 32 µg/mL. Notably, the remaining peptides necessitated concentrations surpassing 64 µg/mL to elicit hemolysis (Table 1). In parallel, cytotoxicity experiments performed on RAW264.7 macrophages demonstrated that most PGLa variants maintained cell viability above 80% across a spectrum of concentrations (Fig. 2A). However, it is pertinent to highlight that DiPGLa-H, at 128 µg/mL, induced a reduction in cell viability to 74.3%, a concentration notably surpassing its average minimum inhibitory concentration (MIC) against pathogens (3.56 µM) (Table 1). These findings collectively underscore the commendable biocompatibility exhibited by all nine PGLa-derived peptides.

**TABLE 1:** MHC, GM_MIC_, and TI values of the engineered peptides

Peptide	MHC^*c*^	GM_MIC_^*a*^			TI^*b*^		
		Gram-negative	Gram-positive	All	Gram-negative	Gram-positive	All
DiPGLa-H (Bios)	>128	2.82	5.66	3.56	45.39	22.61	35.96
DiPGLa-H	>128	2.82	5.66	3.41	45.23	22.61	35.96
kiadin-2	>128	4.76	32	8.98	26.89	4.00	14.25
kiadin-1	128	11.31	22.63	14.25	5.66	2.83	4.49
PGLa-B2	>128	32	64	40.32	4.00	2.00	3.17
PGLa	>128	38.05	64	45.25	3.36	2.00	2.83
PGLa-MW1	128	45.25	64	50.80	1.41	1.00	1.26
PGLa-AM1	128	53.82	64	57.02	1.19	1.00	1.12
PGLa-B1	64	53.82	64	57.02	0.59	0.50	0.56
PGLa-H	>128	128	128	128	2.00	2.00	2.00

^*a*^ The GM of the peptide MICs against bacteria was calculated.

^*b*^ TI is calculated as MHC/GM. Larger values indicate greater cell selectivity.

^*c*^ MHC is the minimum hemolytic concentration that caused 10% hemolysis of hRBCs. Data are representative of three independent experiments. When no detectable hemolytic activity was observed at 128 µg/mL, a value of 256 µg/mL was used to calculate the TI.

The therapeutic index (TI) serves as a pivotal gauge of an antibacterial agent’s cell selectivity and overall clinical applicability. Notably, the TIs of PGLa variants against Gram-negative bacteria significantly surpassed those against Gram-positive bacterial strains (Table 1). Within the cohort of these peptides, DiPGLa-H stood out with the highest TI (TI_all_ = 35.96), a figure that dwarfed the performance of the least effective variant, PGLa-B1 (TI_all_ = 0.56), by a staggering 64-fold (Table 1). Furthermore, when compared with its variants, kiadin-1 (TI_all_ = 4.49) and kiadin-2 (TI_all_ = 14.25), DiPGLa-H’s TI was 8-fold and 2.5-fold superior, respectively. These findings underscore the preeminence of DiPGLa-H among the nine variants (Table 1). Moreover, the substantiation of its superior antibacterial activity against Gram-negative bacteria relative to Gram-positive bacteria was reinforced by additional *in vitro* killing curves. These experiments affirmed that DiPGLa-H manifested a more robust antibacterial activity against *E. coli* in comparison to *S. aureus* (Table 1). At a concentration four times that of its MIC, DiPGLa-H accomplished complete eradication of *E. coli* K88 within 30 min, whereas it required 6 h to achieve a commensurate outcome against *S. aureus* Mu50 (Fig. 2B). These observations further underscore the superior antibacterial activity of DiPGLa-H to Gram-negative bacterial pathogens.

In pursuit of elucidating the antibacterial efficacy of the nine PGLa variants, we embarked on determining their minimum inhibitory concentration (MIC) and minimum bactericidal concentration (MBC) against five pathogenic bacterial strains. MBC values for most peptides closely paralleled their respective MIC values; therefore, the assessment of antibacterial activity predominantly centered around the MIC (Table 2). Our analysis revealed that PGLa exhibited modest antibacterial efficacy against *E. coli* K88 and *Acinetobacter baumannii* 19606, with respective MIC values of 32 µM and 16 µM. In contrast, the MIC of PGLa-H, retaining only the C-terminal 10 amino acids, was greater than 128 µM (Table 2). In stark contrast, DiPGLa-H demonstrated remarkable antibacterial activity, yielding impressive MIC values of 0.96 µM against *A. baumannii* 19606 and 8 µM against *S. aureus* Mu50. The derived peptides kiadin-1 and kiadin-2 (Val-Gly replacements) of DiPGLa-H displayed a reduced antibacterial efficacy (2–8 times lower compared with DiPGLa-H), signifying the pivotal role played by valine in the antibacterial effectiveness of DiPGLa-H (Table 2).

**TABLE 2:** MICs^*a*^ (MBCs^*b*^) (*µ*M) of the engineered peptides against gram-negative and gram-positive bacteria

Peptide	Gram-negative			Gram-positive		
	*E. coli* K88	*E. coli* ATCC25922	*A. baumannii* ATCC19606	*P. aeruginosa* ATCC27853	*S. aureus* ATCC29213	*S. aureus* Mu50
DiPGLa-H (Bios)^*c*^	4 (4)	8 (8)	1 (4)	2 (2)	4 (4)	8 (16)
DiPGLa-H	4 (4)	8 (8)	1 (4)	2 (2)	4 (4)	8 (16)
kiadin-2	4 (16)	8 (16)	4 (4)	4 (8)	32 (32)	32 (32)
kiadin-1	16 (16)	32 (64)	4 (4)	8 (16)	16 (16)	32 (32)
PGLa-B2	32 (64)	64 (64)	8 (8)	>64 (>64)	64 (64)	64 (64)
PGLa	32 (64)	>64 (>64)	16 (16)	64 (64)	64 (64)	>64 (>64)
PGLa-MW1	64 (>64)	>64 (>64)	16 (16)	>64 (>64)	>64 (>64)	>64 (>64)
PGLa-AM1	>64 (>64)	>64 (>64)	32 (32)	64 (>64)	>64 (>64)	>64 (>64)
PGLa-B1	>64 (>64)	>64 (>64)	32 (64)	64 (>64)	64 (64)	64 (64)
PGLa-H	>128 (>128)	>128 (>128)	>128 (>128)	>128 (>128)	>128 (>128)	>128 (>128)

^*a*^ MIC (*µ*M) was determined as the lowest concentration of peptide that inhibited 95% of the bacterial growth. Data are representative of three independent experiments.

^*b*^ MBCs (*µ*M) were determined as the lowest peptide concentration that killed greater than 99.9% of the bacterial cells. The data were derived from representative values of three independent experimental trials.

^*c*^ Bios represents that the peptide is biosynthetic, and other peptides are chemically synthesized.

In elucidating the variances in antibacterial activity among the diverse PGLa variants, we delved into the ability of these peptides to permeabilize the outer and inner membranes of *E. coli* K88. The addition of N-phenyl-1-naphthylamine (NPN), which binds to compromised bacterial outer membranes, facilitated the quantification of the extent of outer membrane damage. The permeabilization of the outer membrane by these nine peptides was concentration-dependent (Fig. 2C). DiPGLa-H, notably, emerged as the leader in terms of outer membrane permeability. PGLa-H failed to exhibit any discernible outer membrane permeability at a concentration of 4 µM, whereas the other eight peptides displayed permeabilities exceeding 50%. DiPGLa-H exerted the most extensive damage at 64 µM (184.4%). For the assessment of inner membrane damage, propidium iodide (PI), which binds to DNA within bacterial cells and emits red fluorescence, was employed. Our observations (Fig. 2D) revealed that DiPGLa-H was most efficacious in damaging the inner membrane at lower concentrations (4 µM) (267%). Conversely, PGLa-H exhibited relatively constant PI-DNA fluorescence intensity at concentrations of 4 µM, 16 µM, and 64 µM, signifying its inability to penetrate the inner membrane of *E. coli* K88 at concentrations below the MIC. The other eight peptides illustrated concentration-dependent effects, attaining peak values at 64 µM. This body of evidence indicates that the robust antibacterial activity of DiPGLa-H may be attributed to its pronounced capacity to disrupt both the bacterial outer and inner membranes.

### The biosynthesized DiPGLa-H exhibits good stability and bacteriostatic activity

**Fig 3:**
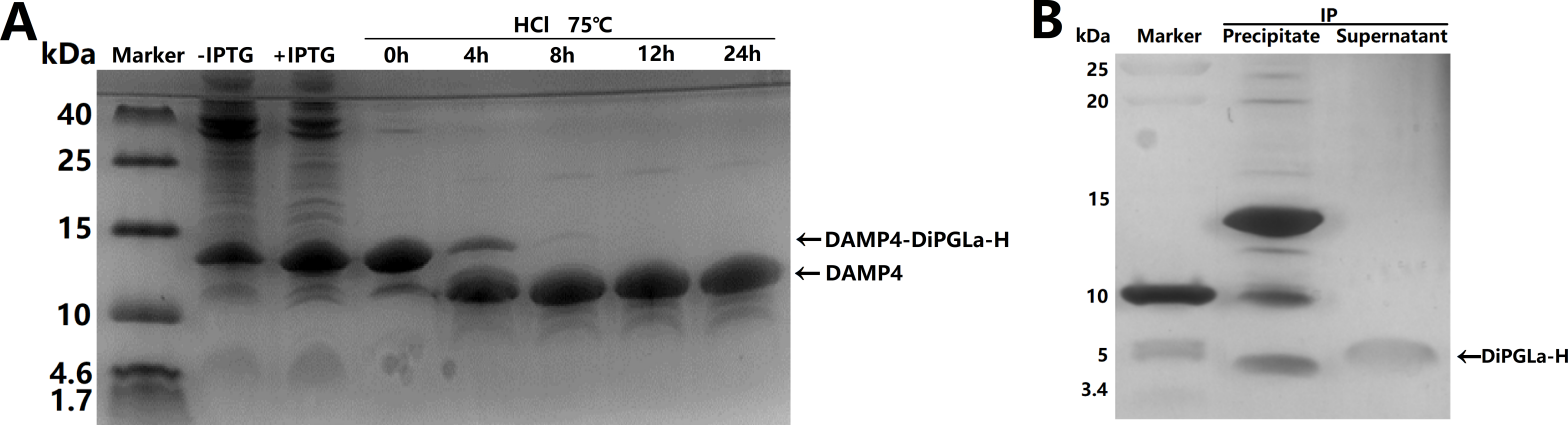
Expression and purification of fusion protein DAMP4-DiPGLa-H and AMP DiPGLa-H. (**A**) Tricine-SDS-PAGE showing the expression and acid cleavage of DAMP4-DiPGLa-H. Total bacterial proteins before and after IPTG induction, and the results of non-chromatographic purification of DAMP4-DiPGLa-H by hydrochloric acid cleavage at 75°C for 0 h, 4 h, 8 h, 12 h, and 24 h are shown. (**B**) The supernatant and precipitate of acid cleavage products of DAMP4-DiPGLa-H treated with isoelectric precipitation (IP), which were showed by Tricine-SDS-PAGE with silver staining.

The inherent cost and practical limitations of chemical synthesis of AMPs have prompted us to explore the biological production of DiPGLa-H. Considering its broad-spectrum antibacterial activity encompassing both Gram-positive and Gram-negative bacteria, the bioproduction strategy necessitated the adoption of a fusion protein approach (15). Our endeavor resulted in the successful expression of a fusion protein, DAMP4-DPS-DiPGLa-H, with a molecular weight of 13.7 kDa in *E. coli* BL21 (DE3) (Fig. 3A, lane 3). Notably, DAMP4 fusion proteins demonstrated favorable solubility and stability, even under conditions of extreme temperature (up to 110°C) and high sodium sulfate concentrations. These attributes facilitated impurity removal through the maintenance of elevated temperatures and sodium sulfate levels. Consequent to these purification protocols, we obtained a pure DAMP4-DiPGLa-H (Fig. 3A, lane 4). Furthermore, the aspartyl-prolinyl peptide bond exhibits considerable instability and is prone to hydrolysis under acidic conditions. We observed most of the fusion protein bands diminished after 4 h at 75°C with HCl treatment. Subsequently, the appearance of acid-digested DAMP4 bands at 11.2 kDa indicated substantial cleavage. Following 8 h of treatment, the fusion protein was entirely cleaved (Fig. 3A). An application of the isoelectric precipitation (IP) method (pH = 6.8) enabled the removal of the DAMP4 tag, yielding highly purified DiPGLa-H (Fig. 3B). The size of the purified DiPGLa-H bands approximated twice the theoretical molecular weight, indicative of oligomerization (Fig. 3B). The bio-produced DiPGLa-H exhibited equivalent antibacterial activity to its chemically synthesized counterpart (Tables 1 and 2).

The stability of peptides is an indispensable facet that underpins their antibacterial efficacy. To this end, we meticulously investigated the sensitivity of bio-produced AMP DiPGLa-H to diverse salt ions, pH levels, and high-temperature conditions (Table S2). Notably, cationic ions were found to variably inhibit the antibacterial activity of DiPGLa-H against *E. coli* K88, resulting in a 2–8 times elevation in MIC values, with Ca^2+^ and Fe^3+^ demonstrating the most profound inhibitory impact (Table S2). The cations exerted a significant inhibitory influence on DiPGLa-H’s antibacterial activity against *S. aureus* Mu50, causing a more than fourfold increase in MIC values (Table S2). DiPGLa-H’s antibacterial efficacy against *E. coli* K88 displayed a lesser degree of susceptibility to high temperature and pH treatments (Table S2). Nonetheless, its antibacterial efficacy against *S. aureus* Mu50 experienced a 4-fold increase or more in MIC when exposed to these adverse conditions.

Biofilm formation constitutes a pivotal factor in enhancing bacterial colonization, infection, and the development of antibiotic resistance. In this context (above 4 µM), DiPGLa-H demonstrated effective inhibition of biofilm formation by *E. coli* K88 and *A. baumannii* ATCC19606, with inhibition at higher concentrations against *P. aeruginosa* ATCC27853 and *S. aureus* Mu50 (Fig. S1A). Subsequent assessment of biofilm elimination potential was conducted by allowing biofilms to form over 2 days, followed by treatment with DiPGLa-H. These results unveiled that elevated concentrations of DiPGLa-H (above 16 µM) effectively eradicated biofilms of *E. coli* K88, *A. baumannii* ATCC19606, and *S. aureus* Mu50 (Fig. S1B). Nevertheless, the biofilm elimination capacity of DiPGLa-H appeared less pronounced against *P. aeruginosa* ATCC27853 at similar concentrations.

### DiPGLa-H exerts bacteriostatic activity by disrupting bacterial cell membrane integrity and inducing ROS production

**Fig 4:**
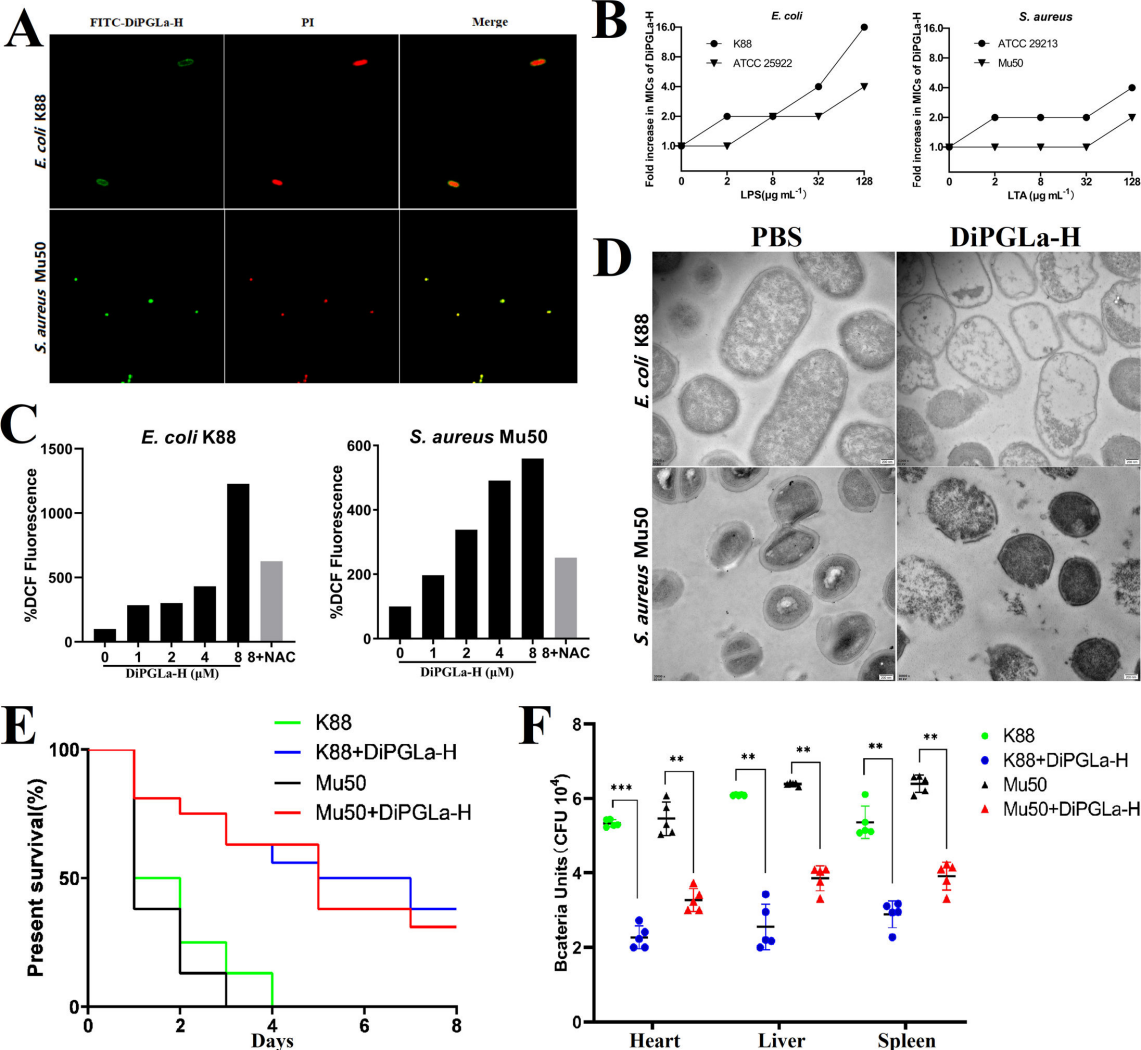
Bactericidal mechanism and *in vivo* treatment of DiPGLa-H. (**A**) Super-resolution microscopy image analysis of *S. aureus* Mu50 and *E. coli* K88 treated with FITC-labeled DiPGLa-H and PI. (**B**) Effects of exogenous addition of different doses of LPS and LTA on the antibacterial activity of DiPGLa-H. (**C**) ROS changes of *E. coli* K88 and *S. aureus* Mu50 treated with 0–8 μM DiPGLa-H, in which NAC is an antioxidant. (**D**) Transmission electron microscopy (TEM) micrographs of *E. coli* K88 and *S. aureus* Mu50 before and after DiPGLa-H treatments. (**E**) The model of abdominal infection in mice, the survival rate of mice before and after DiPGLa-H treatment, 16 mice in each group. (**F**) After infection, the organs of the mice were shaved and homogenized, and the plate was coated to calculate the number of colonies, with 5 mice in each group.

In our quest to ascertain the binding sites of DiPGLa-H on the bacterial cell surface, we harnessed fluorescein isothiocyanate (FITC)-labeled DiPGLa-H. The outcome indicated that green fluorescence emanating from FITC-DiPGLa-H extended across the entire cell surfaces of both *E. coli* K88 and *S. aureus* Mu50 (Fig. 4A); furthermore, propidium iodide (PI) staining revealed the aggregation of nucleic acids within the cells (Fig. 4A). This concurrence of green fluorescence and PI staining substantiates the binding of DiPGLa-H to the cell surface of *E. coli* K88 and *S. aureus* Mu50, thereby affirming its antibacterial modus operandi of disrupting the integrity of bacterial cell membranes. In another way, lipopolysaccharide (LPS) and lipoteichoic acid (LTA) represent the principal constituents of the outer membrane in Gram-negative bacteria and the cell wall in Gram-positive bacteria, respectively. Not only do these molecules constitute the initial hurdle faced by AMPs in their quest to combat bacterial pathogens, but they also carry negatively charged phospholipid groups. In our experiments, we externally introduced differing concentrations of LPS to examine the impact on DiPGLa-H’s antibacterial activity against *E. coli* K88 (Fig. 4B). These investigations unveiled a significant reduction in DiPGLa-H’s antibacterial activity with the increasing addition of external LPS, resulting in a 16-fold reduction against *E. coli* K88 (Fig. 4B). A similar trend was observed with LTA against *S. aureus*, causing a 2-fold to 4-fold decrease in activity with the addition of 128 µg/mL LTA (Fig. 4B). These outcomes underscore the capacity of the cationic AMP DiPGLa-H to bind to negatively charged LPS and LTA, thereby leading to a substantial diminution in its antibacterial activity upon the external introduction of these molecules. The quantification of reactive oxygen species (ROS) levels demonstrated a dose-dependent relationship with the concentration of DiPGLa-H in both *E. coli* and *S. aureus* (Fig. 4C). The treatment with 8 µM DiPGLa-H markedly elevated ROS production by 11.3-fold in *E. coli* K88 and 4.6-fold in *S. aureus* Mu50 compared with untreated groups. The addition of the antioxidant N-acetylcysteine (NAC) significantly attenuated the fluorescence intensity exhibited by the 8 µM DiPGLa-H-treated groups (Fig. 4C). This attenuation stands as a testament to the excessive generation of ROS consequent to DiPGLa-H treatment, further intensifying bacterial mortality.

To visually elucidate the impact of DiPGLa-H on bacterial cell morphology, we conducted observations under transmission electron microscopy following DiPGLa-H treatment. These examinations disclosed significant deviations in the structural integrity of *E. coli* K88 and *S. aureus* Mu50 upon DiPGLa-H exposure. The consequences included marked separation between the inner and outer membranes of *E. coli* K88, accompanied by notable wrinkling, vesiculation, porosity on the cell surface, and the release of cellular contents. A parallel impact was observed in *S. aureus* Mu50, with the cell wall appearing disrupted, leading to the formation of pores on the surface, an indistinct demarcation between the cell wall and cell membrane, sparse cytoplasmic distribution, and substantial leakage (Fig. 4D). Consequently, the findings from transmission electron microscopy affirm that DiPGLa-H treatment triggers significant alterations in bacterial cell morphology, destabilizes the integrity of the cell wall and cell membrane, elicits the release of bacterial contents, and ultimately leads to bacterial demise. The experimental results of mouse abdominal infection showed that all mice infected by intraperitoneal injection of bacterial solution K88 and Mu50 died within 4 days, whereas the survival rate of mice was increased by 38% and 31% after DiPGLa-H treatment, respectively (Fig. 4E; Table S3). In addition, after the mice were infected with K88 and Mu50, the loads of *E. coli* and *S. aureus* were collected and calculated in the heart, liver, and spleen, and the loads of bacteria were significantly reduced in the DiPGLa-H treatment group, in which the treatment group with *E. coli* was able to reduce the bacterial loads in the organs a 1,000-fold and that of *S. aureus* was able to reduce the bacterial loads in the organs a 100-fold (Fig. 4F). This suggests that DiPGLa-H is an AMP with potential application in mice.

## DISCUSSION

Currently, AMPs are gaining increasing attention as a novel class of antimicrobial agents, but several factors limit their development (16). For example, natural AMPs like PGLa and magainin exhibit relatively low antimicrobial activity and a narrow spectrum of activity (17). Although the bee venom peptide melittin has strong antimicrobial activity, it also exhibits higher hemolytic activity and cytotoxicity (18). Therefore, systematic comparison of a certain AMP family and screening for variant with excellent *in vitro* and *in vivo* anti-infective activity has good theoretical and practical values. In this study, a series of variants were synthesized based on PGLa. The natural variants of PGLa included PGLa, PGLa-B2, PGLa-MW1, PGLa-B1, PGLa-AM1, and PGLa-H, whereas the designed PGLa variants included DiPGLa-H, kiadin-1, and kiadin-2. Based on a systematic comparison, it is proved variant DiPGLa-H exhibits the most outstanding bacteriostatic and clinical applicability among nine variants. DiPGLa-H was further biosynthesized and exhibited good stability and bacteriostatic activity by disrupting bacterial cell membrane integrity and inducing ROS production.

Arginine and lysine are responsible for the cationic nature of AMPs at physiological pH, and they can interact with the surface of bacteria through electrostatic interactions (19, 20). Many AMPs have a net positive charge of 4–6, and an excess or deficiency of positive charges can lead to reduced antimicrobial efficiency and cell selectivity. The designed variants of PGLa carry six charges, with significantly better bacteriostatic activity (MIC = 3.56 ~ 14.25 µM) than PGLa natural variants (MIC = 40.32 ~ 128 µM). The positive charges in PGLa natural peptides range from 3 to 4, indicating that positive charges are prerequisites for antimicrobial activity (21). In another way, researchers have found that hydrophobicity can modulate the antimicrobial efficiency and specificity of AMPs (22, 23). Among the PGLa designed variants, DiPGLa-H, with 43.8% hydrophobic values, exhibited the best antimicrobial activity, whereas the designed peptides kiadin-1 and kiadin-2 showed slightly lower antimicrobial activity, with hydrophobic values of 37.7%, lower than DiPGLa-H. Increasing hydrophobicity beyond a certain level may lead to increased mammalian toxicity. At 128 µg/mL concentration, DiPGLa-H demonstrated cytotoxicity, although this concentration is much lower than its comprehensive antimicrobial activity (GM = 35.96). PGLa-B2 had the highest hydrophobicity (46.4%) among PGLa natural variants and exhibited the strongest overall antimicrobial activity among the natural peptides.

Many AMPs with less than 30 amino acids are amphipathic, possessing both hydrophilic and hydrophobic structural domains (24, 25). The relative hydrophobic moment reflects the level of amphipathicity of the peptide. Designed PGLa variants all maintained a high level of amphipathicity (0.406–0.477), whereas natural peptides, except for PGLa-H, had lower hydrophobic moment values (0.270–0.376). Although PGLa-H had the best amphipathic arrangement (0.551), it exhibited the weakest antimicrobial activity, which may be attributed to its shorter amino acid sequence and low positive charge (only three positive charges). The average antimicrobial activity of DiPGLa-H approximately 18 times higher than PGLa-H. The helical wheel projection of DiPGLa-H showed an imperfect amphipathic pattern, which may favor membrane perforation (26). Membrane integrity experiments revealed that PGLa-H had the weakest membrane-disrupting ability, suggesting that amphipathicity level is not necessarily directly correlated with antimicrobial activity, whereas the size and charge of AMPs also need to be considered. The kiadin-1 and kiadin-2 peptides, which were designed by Val-Gly substitution of DiPGLa-H, showed a slight reduction in antimicrobial activity (GM_ALL_ values more than double that of DiPGLa-H), particularly with a significant reduction in antibacterial activity against *S. aureus* Mu50. This was inconsistent with the results of previous studies (8), suggesting the antibacterial activity of AMPs requires multiple verifications to facilitate their ultimate application.

The antimicrobial effect of the PGLa variants is generally better against Gram-negative bacteria than Gram-positive bacteria, which might be attributed to the thicker cell wall of Gram-negative bacteria. The cationic AMPs preferentially interact with the anionic components of the cell wall of Gram-negative bacteria (such as WTA and LTA) (27–30), whereas the anionic components LPS of Gram-negative bacteria are more prone to binding AMPs. Our experiments illustrated concentration-dependent binding of DiPGLa-H to LPS and LTA, suggesting that its inhibitory activity may involve initial binding to anionic components on the bacterial surface. Permeability experiments showed that DiPGLa-H had the highest membrane permeability, reaching approximately 80% at a concentration of 1× MIC. Aggregated peptide concentration beyond a critical threshold-initiated interaction with the lipid membrane, forming a stable secondary structure, disrupting surface tension, inducing membrane depolarization, and causing cell lysis and death (31). TEM results corroborated DiPGLa-H-induced bacterial membrane damage. ROS experiments demonstrated elevated intracellular ROS in bacteria treated with DiPGLa-H, contributing to intracellular oxidative damage. In conclusion, our findings propose a multifaceted inhibitory mechanism of DiPGLa-H on bacterial intracellular and cellular membranes (32, 33).

Assurances for clinical applications include the stability of AMPs at different environmental concentrations and their impact on the bacterial biofilms (34, 35). Changes in salt ion concentrations, pH, and high-temperature treatments did not significantly diminish DiPGLa-H’s bacteriostatic ability against *E. coli*, but reductions were observed against *S. aureus* Mu50. DiPGLa-H demonstrated effective inhibition of biofilm formation, and elevated concentrations of DiPGLa-H (above 16 µM) effectively eradicated biofilms of *E. coli*, *A. baumannii*, and *S. aureus*.

### Conclusions

In summary, we employed solid-phase synthesis to generate nine PGLa variants, systematically exploring the impact of various structural parameters. Among them, the designed peptide DiPGLa-H exhibits the most outstanding anti-infective activity. Across the peptide cohort, all designed peptides consistently conferred superior bacteriostatic activity, indicating that artificial selection expedited the evolutionary trajectory of natural peptides. Within a defined parameter space, we identified positive charge (prefer lysine residue) as prerequisites for enhancing AMP activity. Moreover, hydrophobicity emerged as a crucial determinant influencing the antimicrobial efficacy. Mechanistically, our investigation revealed that DiPGLa-H effectively penetrated bacterial cell membranes, instigating heightened intracellular ROS production, thereby exacerbating bacterial demise. Furthermore, the observed stability of DiPGLa-H and its robust resistance to biopermeability present pivotal assurances for clinical applications. The cost-effective production of DiPGLa-H through heterologous expression and purification in *E. coli* holds significant promise for future applications in livestock production.

## MATERIALS AND METHODS

### Materials

The PS-kiadin-HH peptide (PSKIAKVALKALKIAKGALKALHH-NH_2_) with a molecular weight of 2607.11 Da, was custom synthesized by Sangon Biotech (Shanghai) with 97.71% purity. Unless otherwise specified, all biochemical reagents, buffers, and culture media were procured from Sangon Biotech (Shanghai). Restriction endonuclease and T4 DNA ligase were purchased from New England Biolabs (Beijing Ltd.). The strains and plasmids utilized in this study are detailed in Table S4, whereas the primers are listed in Table S5. *E. coli*, *A. baumannii*, *P. aeruginosa,* and *B. subtilis* in this experiment were cultured in LB medium. *S. aureus* were cultured in TSB.

### Synthesis and characterization of peptides

Nine PGLa variants were found on the website http://dramp.cpu-bioinfor.org/ and related literatures. The engineered peptides were synthesized by Sangon Biotech (Shanghai, China), and their fidelity was determined by matrix-assisted laser desorption/ionization time-of-flight mass spectrometry (MALDI-TOF MS; Linear Scientific Inc., USA). The peptide purity (>95%) and retention time were tested by reverse-phase high-performance liquid chromatography (HPLC) with a column of GS-120–5-C18-BIO 4.6 × 250 mm, 220 nm, 10 µL volume using a nonlinear water/acetonitrile gradient that contained 0.1% Trifluoroacetic at a flow rate of 1.0 mL/min. The charge and hydrophobic moment were calculated online using the HeliQuest analysis website (http://heliquest.ipmc.cnrs.fr/cgi-bin/ComputParamsV2.py). The 3D structure projection was predicted online with I-TASSER (http://zhanglab.ccmb.med.umich.edu/I-TASSER).

### CD spectroscopy

CD spectra of the peptides were recorded with a J-820 spectropolarimeter (Jasco, Tokyo, Japan) at 25°C. The spectra were measured in 10 mM PBS, 30 mM SDS, 10 mM DPC, and 50% TFE (the final concentration of the peptide was 150 µM). The CD spectra were recorded at a wavelength of 200–250 nm, and the data are expressed as mean residue ellipticity. The acquired CD signal spectra were then converted to the mean residue ellipticity using the following equation: θ_M_= (θ_obs_.1000)/(c.l.n), where θ_M_ indicates the residue ellipticity (deg cm^2^ dmol^−1^), θ_obs_ represents the observed ellipticity corrected for the buffer at a given wavelength (mdeg), c is the peptide concentration (mM), l is the path length (mm), and n is the number of amino acids.

### Biocompatibility assays

Cytotoxicity and hemolysis assays were performed as described previously (36). Briefly, RAW264.7 cells and pig red blood cells were treated with varying concentrations of peptides. The cytotoxic and hemolytic effects were quantified using MTT and hemoglobin release assays, respectively. Full experimental details are provided in the Supplementary Materials.

### Antimicrobial activity assays

The antibacterial activity was evaluated using a standard MIC and MBC assay according to the National Committee for Clinical Laboratory Standards (NCCLS). MIC, MBC assay, and time-kill curves for the optimal peptide are provided in the Supplementary Materials.

### Expression and purification

We designed and synthesized the target gene for DAMP4-DiPGLa-H (Sangon Biotech, Shanghai, China), ligated the target gene to the pET-28a plasmid by T4DNA ligase (Thermo, America), and then transformed it into the expression host BL21 (DE3) and incubated in LB medium at 16°C for 16 h induction using IPTG (Sangon Biotech, Shanghai, China) at a final concentration of 0.5 µM. Cell sediments were collected by centrifugation (4,000 × *g*, 4°C, 20 min) and stored at −20°C. All bacterial cell cultures were supplemented with 50 µg/mL kanamycin sulfate. The 0.8M Na_2_SO_4_ is then added and held in a water bath at 90°C for 30 min. Separation of the soluble DAMP4var-pexiganan component from the precipitated contaminants was achieved by centrifugation at 39,000 G for 10 min, which was poured into a new beaker to ensure that the pellet was not disturbed. Hydrochloric acid (HCl) was then added to an aliquot (45 mL) of the supernatant until the pH reached pH 3 and then diluted five times with Milli-Q water. The solution becomes cloudy and is allowed to stand for 30 min, then centrifuged at 39,000 G for 10 min to separate the supernatant from the precipitated fraction. The white precipitate is resuspended in 10 mL of Milli-Q water. HCl was added to 10 mL of thermally purified DAMP4-DiPGLa-H aqueous solution to a final HCl concentration of 60 mM, followed by acid lysis at 60°C for 48 h with intermittent sampling. The pH of the white precipitated suspension species was adjusted to 6.8 to the isoelectric point of DAMP4, left for 30 min at 37°C, then centrifuged at 39,000 G for 10 min, and the supernatant was removed.

Peptides smaller than 10 kD were separated by configuring Tricine-SDS-PAGE using a small molecular weight protein gel preparation kit (Sangon Biotech, C641100-0150, Shanghai, China), and the size of the AMPs was detected using silver staining. Sample preparation, staining, and destaining were performed as recommended by the manufacturer.

### Heat, pH, and salt sensitivity assays

To assess the stability of the peptides in physiological conditions, the peptides were incubated with *E. coli* K88 and *S. aureus* Mu50 in the presence of salts (150 mM NaCl, 4.5 mM KCl, 6 µM NH_4_Cl, 8 µM ZnCl_2_, 1 mM MgCl_2_, 4 µM FeCl_3_, and 2.5 µM CaCl_2_) in accordance with a previously reported protocol. To determine the thermal stability of the peptides, the peptides were pretreated at 100°C for 30 min, 60 min, and 90 min and cooled on ice for 10 min. The effect of acid and base on the antimicrobial activity was measured by treating the peptides for 2 h at pH of 2, 4, 10, and 12 in 10 mM sodium phosphate buffer adjusted with HCl or NaOH.

### Detection of anti-biofilm ability

The anti-biofilm activity of DiPGLa-H was evaluated using a crystal violet staining method. Briefly, 100 µL of bacterial suspension at 1 × 10^6^ CFU/mL was incubated with DiPGLa-H (0–64 µM) for 48 h. The biofilm formed was stained with 1% crystal violet, and the absorbance was measured at 595 nm. The biofilm formation and mature biofilm removal assays were conducted. For biofilm formation, a bacterial suspension was incubated with DiPGLa-H, and biofilm content was quantified. To assess mature biofilm removal, pre-formed biofilms were treated with DiPGLa-H for 24 h, followed by quantification. Detailed Protocol in Supplementary Materials.

### Membrane permeabilization

The outer and inner membrane integrity of bacterial cells treated with peptides was assessed using NPN and PI staining, respectively. Full details are provided in the Supplementary Materials.

### Fluorescence imaging

FITC-labeled peptides and PI (Sigma, USA) were used to localize the site of action of the peptides on conventional and super-resolution fluorescence microscopes. *E. coli* K88 and *S. aureus* Mu50 were grown overnight in LB and TSB medium, respectively, and shaken (200 rpm) for 4 h at 37°C in a fresh medium. Bacterial cells were then harvested by centrifugation at 1,000 *× g* for 10 min, washed three times with PBS buffer, and resuspended to an OD600 nm of 0.2. FITC-labeled peptides were added to the bacterial suspension at MIC value concentrations and shaken (150 rpm) at 37°C for 1 h. PI dye was added to the resuspended bacterial solution to a final concentration of 10 µg/mL and incubated for 30 min. Cells were washed three times with PBS to remove unbound PI, and 5 µL of bacterial solution was spread on the center of a glass slide and covered with a coverslip. Fluorescence images were acquired on a bio-laser confocal microscope (Leica TCS SP8, Germany) and LAS X system (Wetzlar, Germany) with excitation wavelengths of 488 and 535 nm for PI and FITC, respectively.

### LPS and LTA binding assay

LPS (Sigma, USA) and LTA (Sigma, USA) were dissolved in water at a concentration of 5 mg/mL. Fifty microliters of DiPGLa-H with a concentration gradient of 1–64 μM and 50 µL of LPS or LTA master mix with different concentration gradients were added to the 96-well plate in 2-fold dilution, and finally, 100 µL of diluted *E. coli* K88 or *S. aureus* Mu50 was added, incubated at 37°C in a constant temperature incubator for 24 h. The MIC was determined by absorbance at 492 nm using an enzyme marker as the lowest peptide concentration that inhibited 95% of the bacterial growth.

### ROS generation

2′,7′-Dichlorofluorescin diacetate (DCFH-DA) was used to determine the intracellular production of ROS as previously described. *S. aureus* Mu50 and *E. coli* K88 cells were diluted until OD600 = 0.4 and incubated in different concentrations of peptide at 37°C for 1 h. Next, the mixed solution was incubated with 10 µM DCFH-DA at 37°C for 1 h. The fluorescence (excitation wavelength = 488 nm, emission wavelength = 525 nm) was monitored with an Infinite 200 pro fluorescence spectrophotometer (Tecan, China). Each test was measured in triplicate and tested twice.

### TEM

Pick *S. aureus* Mu50 and *E. coli* K88 monoclonal shaking bacteria overnight, secondary activation of the bacteriophage in the transfer of 500 µL to 50 mL of fresh liquid medium, shaker 250 rpm, 37°C incubation 2 h to log phase. The bacterial broth was centrifuged at 4°C, 3000 rpm for 10 min, and washed twice with pre-cooled PBS at 4°C, 3000 rpm for 10 min. Add PBS to the washed bacteriophage, blow well, measure OD600nm, and dilute the bacteriophage with PBS to 10^8^ CFU/mL. Mix the MIC value concentration of DiPGLa-H with the bacteriophage and incubate at 37°C incubator at 250 rpm for 1 h. Centrifuge the samples at 4°C, 3000 rpm for 5 min, and wash twice with PBS; use 500 µL of 2.5% solution. The bacteria were resuspended with 500 µL of 2.5% glutaraldehyde and fixed overnight at 4°C. Samples were washed three times with PBS for 15 min each, then fixed with starvation acid for 2–4 h. Samples were washed three times with PBS for 10 min each, dehydrated with different gradients (30%, 50%, 70%, 80%, 90%, and 100%) of ethanol, two times for each gradient, then transferred to a 1:1 mixture of anhydrous acetone and epoxy resin for 30 min, and then transferred to pure epoxy resin and incubated at constant temperature overnight. Finally, the specimens were sectioned with an ultrathin sectioning machine, stained with UO2 acetate and lead citrate, and visualized using TECNAI G2 SPIRIT BIO (FEI, America).

### Mouse abdominal infection model

Six-to-eight-week-old C57BL/6 male mice were selected for the experiment and housed in cages under specific pathogen-free conditions with water and laboratory mouse chow. Each strain was divided into three groups, and each group and the second group were required to be injected intraperitoneally with a lethal amount of bacterial fluid with a working fluid concentration of 64 µg/mL of DiPGLa-H, and the third group was injected with PBS. 16 mice in the first group were observed for the mortality rate, and five mice in the second group were uniformly put to death after 1 day of infection, and the mice were dissected to remove the livers, kidneys, and other organs. Then homogenized and serially diluted with PBS to 10^7^ times. 100 µL of the gradient dilution was applied to the TSA plate, which was subsequently incubated at 37°C overnight and counted the next day for survival analysis using GraphPad Prism software. The protocol and use of animals were approved by the Institutional Animal Care and Use Committee of the Northwest A&F University.

## Data Availability

The data that support the finding of this study are available in the supplemental material of this article.

## SUPPLEMENTAL MATERIAL

Supplemental material aem.02062-24-s0001.docx Figure S1, Tables S1 to S5, and supplemental methods.

ASM does not own the copyrights to Supplemental Material that may be linked to, or accessed through, an article. The authors have granted ASM a non-exclusive, world-wide license to publish the Supplemental Material files. Please contact the corresponding author directly for reuse.
