# Supplementary material for: Antimicrobial peptide DiPGLa-H exhibits the most outstanding anti-infective activity among the PGLa variants based on a systematic comparison
Source: Appl Environ Microbiol. 2025 Feb 5;91(3):e02062-24. doi: 10.1128/aem.02062-24 (PMC11921344; doi:10.1128/aem.02062-24)
Supplement: Supplemental material — Figure S1, Tables S1 to S5, and supplemental methods. [file aem.02062-24-s0001.docx]

**Supplementary Figure**


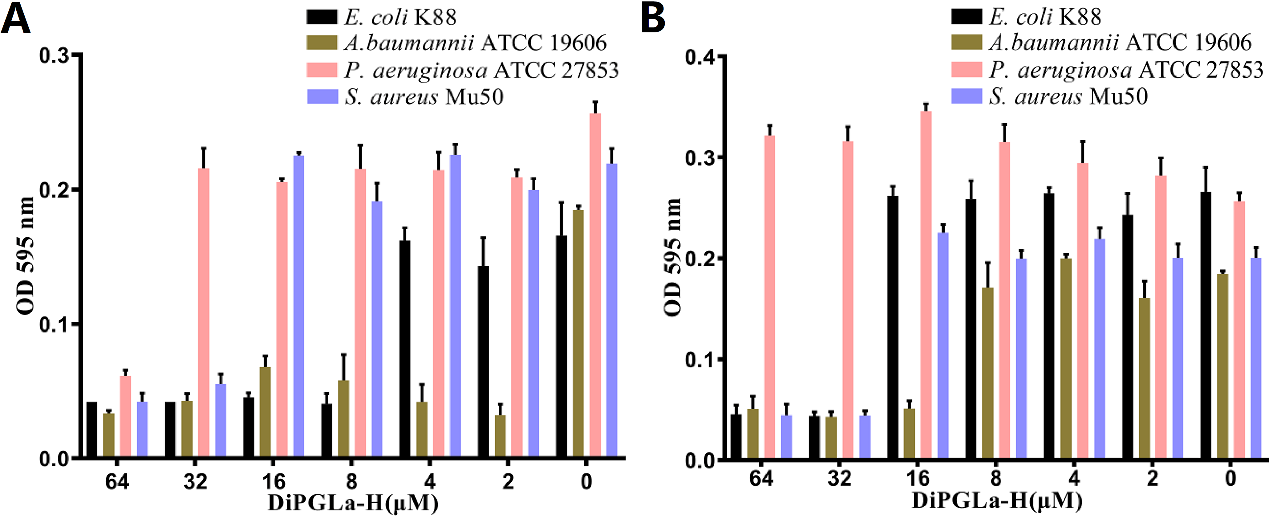


**Figure S**1: The effect of antimicrobial peptide DiPGLa-H on the formation (A) and eradication (B) ability of pathogenic bacterial biofilms at 0-64 μM.

**Supplementary Tables**

**Table S1. Peptide design and their key physicochemical parameters**

| peptide | theoretical MW^a^ | measured MW | net charge | H^b^ | *μ*Hrel^c^ | Purity (%) |
| --- | --- | --- | --- | --- | --- | --- |
| DiPGLa-H | 2090.75 Da | 2089.78 Da | +6 | 0.438 | 0.422 | 96.01% |
| kiadin-2 | 2048.67 Da | 2048.20 Da | +6 | 0.377 | 0.406 | 98.62% |
| kiadin-1 | 2048.67 Da | 2047.95 Da | +6 | 0.377 | 0.477 | 97.71% |
| PGLa-B2 | 1956.42 Da | 1955.45 Da | +3 | 0.464 | 0.365 | 96.88% |
| PGLa | 1969.46 Da | 1969.00 Da | +4 | 0.363 | 0.353 | 96.08% |
| PGLa-MW1 | 1986.44 Da | 1985.47 Da | +3 | 0.457 | 0.376 | 96.66% |
| PGLa-AM1 | 2070.57 Da | 2069.60 Da | +4 | 0.355 | 0.281 | 96.08% |
| PGLa-B1 | 2114.62 Da | 2113.65 Da | +4 | 0.382 | 0.270 | 95.88% |
| PGLa-H | 1054.38 Da | 1053.40 Da | +3 | 0.438 | 0.551 | 97.25% |

^a^Molecular weight (MW) was confirmed by mass spectroscopy (MS).

^b^Hydrophobicity (H) values means the total hydrophobicity (sum of all residue hydrophobicity indices) divided by the number of residues, and they were calculated from <http://heliquest.ipmc.cnrs.fr/cgi-bin/ComputParams.py>.

^c^Relative hydrophobic moment (μHrel) values were employed to analyze the level of amphipathicity of all peptides and were calculated from <http://heliquest.ipmc.cnrs.fr/cgi-bin/ComputParams.py>.

**Table S2. MIC values of DiPGLa-H against *E. coli* K88 and *S. aureus* Mu50 under different environmental conditions**

|  | **MIC(μM)** | |
| --- | --- | --- |
|  | ***E. coli* K88** | ***S. aureus* Mu50** |
| DiPGLa-H (Without treatments) | 4 | 8 |
| NaCl (150mM) | 16 | >32 |
| KCl (4.5mM) | 8 | >32 |
| NH_4_Cl (6μM) | 8 | >32 |
| MgCl_2_ (1mM) | 16 | >32 |
| CaCl_2_ (2.5μM) | >32 | >32 |
| ZnCl_2_ (8μM) | 8 | >32 |
| FeCl_3_ (4μM) | >32 | >32 |
| pH 2 | 8 | >32 |
| pH 4 | 8 | >32 |
| pH 10 | 8 | 32 |
| pH 12 | 8 | 32 |
| Heat 100℃ (30min) | 8 | >32 |
| Heat 100℃ (60min) | 8 | >32 |
| Heat 100℃ (90min) | 8 | >32 |

**Table S3.** Statistics for Figure 3E

| Days | K88 | K88+DiPGLa-H | Mu50 | Mu50+DiPGLa-H |
| --- | --- | --- | --- | --- |
| 0 | 16 | 16 | 16 | 16 |
| 1 | 8 | 13 | 7 | 12 |
| 2 | 3 | 12 | 2 | 11 |
| 3 | 0 | 10 | 0 | 10 |
| 4 | 0 | 9 | 0 | 8 |
| 5 | 0 | 9 | 0 | 6 |
| 6 | 0 | 9 | 0 | 6 |
| 7 | 0 | 9 | 0 | 5 |
| 8 | 0 | 9 | 0 | 5 |

The data in the table are the number of mice that survived.

**Table S4.** Strains and plasmids used in this study

| **Strains/Plasmids** | **Description** | **Sources** |
| --- | --- | --- |
| **Strains** |  |  |
| DH5α | *Escherichia coli* | Lab stock |
| BL21 (DE3) | *Escherichia coli* | Lab stock |
| ATCC25922 | *Escherichia coli* | Lab stock |
| K88 | *Escherichia coli* | Lab stock |
| BL21 (DE3)-pET28a-DAMP4-DPS-DiPGL-H | *Escherichia coli* | Lab stock |
| ATCC19606 | *Acinetobacter baumannii* | Lab stock |
| ATCC27853 | *Pseudomonas aeruginosa* | Lab stock |
| Mu50 | *Staphylococcus aureus MRSA* | Lab stock |
| ATCC29213 | *Staphylococcus aureus* | Lab stock |
| **Plasmids** |  |  |
| pET28a | *E. coli* expression vector Kan^R^, T7 promoter | This study |
| pET28a-DAMP4-DPS-DiPGLa-H | *E. coli* expression vector Kan^R^, T7 promoter with *DAMP4-DPS-* *DiPGLa-H* gene | This study |

**Table S5**. Sequences of primers used in this study

| **Primer** | **Sequence (5’ to 3’)** |
| --- | --- |
| T7-F | TAATACGACTCACTATAGGG |
| T7-R | TGCTAGTTATTGCTCAGCGG |

**Supplementary experimental methods**

**Biocompatibility Assays.** The murine macrophage cell line RAW264.7 was used to determine the cytotoxicity of the peptides by the 3-(4,5-dimethyl-2-thiazolyl)-2,5-diphenyl-2H-tetrazolium bromide (MTT) assay, and healthy pig red blood cells (hRBCs) were used to evaluate the hemolytic rates of the peptides as previously described. Briefly, for the MTT assay, 1.0−2.0×10^4^ cells/well were inoculated into 96-well plates and were interacted with various peptides (2−128 μg/mL) for 18−24 h at 37°C in 5% CO_2_. Subsequently, the cell culture was further incubated with MTT (0.5 mg/mL) for 4 h at 37 °C. The supernatant was discarded, and the formazan crystals were dissolved in 150 μL of dimethylsulfoxide while the solution was further measured using a microplate reader (Varioskan Flash; Thermo, America) at an OD of 570 nm.

A healthy pig provided a 1 mL sample of fresh hRBCs that was collected and resuspended in PBS (pH 7.4) to obtain a dilution solution of ∼1% (v/v) erythrocytes. Next, the mixtures of equal volumes of hRBC solution and the peptides at different concentrations (1−128 μg/mL) were incubated for 1 h at 37 °C. The mixtures were centrifuged (1000g, 10 min), and then the supernatants were transferred to a new 96-well plate to assess the release of hemoglobin at 576 nm using a Thermo Scientific Microplate Reader (Varioskan Flash; Thermo, America). Additionally, untreated hRBCs served as the negative control (0% hemolysis) and 0.1% Triton X-100 treatment served as the positive control (100% hemolysis). The peptide concentration that caused a hemolytic rate>10% is considered the MHC. The percent hemolysis was calculated via the following formula

Percent hemolysis = [(A-A_0_ )/(A_t_-A_0_)]×100

where A represents the absorbance of the peptide sample at 576 nm, and A_0_ and At represent 0 and 100% hemolysis, determined in 10mM PBS and 0.1% Triton X-100, respectively. At least three independent experiments were conducted for the biocompatibility assays and three technical replicates were used in each independent experiment.

**Antimicrobial Activity Assays.** The antibacterial activity of the peptides was determined using a method designed from the National Committee for Clinical Laboratory Standards (NCCLS) in the United States. All the strains used in the experiment are preserved in the laboratory. Briefly, log phase bacterial cells were diluted in MHB to a final concentration of 0.5-1x10^5^ CFU/mL. Subsequently, 50μL of different concentrations of polypeptides were incubated with 50 μL of bacterial solution in sterile 96-well plates. In the minimum inhibitory concentration (MIC) assay, the plate was incubated at 37°C for 24 h, and the minimum inhibitory concentration (MIC) was determined by the absorbance at 492 nm of the microplate reader, and the minimum inhibitory concentration (MIC) inhibited 95% of bacteria grow. Determination of minimum bactericidal concentration: the plate was incubated at 37°C for 4 hours, 50 μL of the mixture was taken out of each well, diluted with an appropriate amount of PBS, smeared on the agar plate, and cultured overnight. Colonies were counted and the initial CFU/well was retrospectively calculated the following day. The lowest concentration of peptide that kills 99.9% of bacterial cells is the minimum bactericidal concentration (µM), Each test was replicated at least 3 times.

The bactericidal curve of the optimal peptide was further determined. The bactericidal curve of DiPGLa-H against *E. coli* K88 and *S. aureus* Mu50, firstly pick the single clone of *E. coli* K88 and *S. aureus* Mu50 to inoculate into 2mL of TSB, shake and culture at 37°C overnight, then take 100 μL of overnight bacteria They were added to the corresponding conical flasks containing 10mL of TSB, and then DiPGLa-H with a final concentration of 1, 2, and 4 times the MIC concentration was added, respectively, and shaken at 37°C for incubation at 0h, 0.5h, 1h, 1.5h, 2h, 4h, 6h, take 10 μL of gradient dilution and drop it on the MHA-free plate, seal and invert at 37°C for 12-16h, calculate the number of viable bacteria according to the dilution ratio according to the above method, and draw a sterilization curve. Each test was replicated at least 3 times.

**Detection of anti-biofilm ability.**

To test the ability of DiPGLa-H to form biofilm on the test bacteria, a single colony was inoculated in MHB medium and incubated overnight at 37 °C and 180 r/min, and 1% of the bacterial suspension was taken and continued to be amplified and incubated for 2 h. The concentration of the bacterial suspension was adjusted to 0.5 maixicity by OD600, and the MHB medium was continued to be diluted 100 times to 1×10^6^ CFU/mL, and in each well of a 96-well plate Add 100 μL of MHB gradient dilution of DiPGLa-H, then add 100 μL of the above bacterial suspension, incubate at 37℃ in a humid environment in a constant temperature incubator for 48h, then wash 2 times with PBS to remove unadsorbed bacteria, fix with 4% paraformaldehyde for 30 min, discard the fixative, wash with PBS and air dry naturally. Add 100 μL of 1% crystalline violet staining solution to each well at room temperature and stain for 30 min. Aspirate the crystalline violet staining solution, wash with PBS until colorless, and air dry in an ultra clean bench. The biofilm content was analyzed by measuring the absorbance value at 595 nm, and anhydrous ethanol was used as a blank control.

In order to systematically investigate the effect of DiPGLa-H on the removal of mature biofilm, the prepared bacterial suspension was added to 96-well cell culture plates with 100μL per well, incubated in a constant temperature incubator at 37℃ for 72h, then the 96-well plates were removed, the supernatant was discarded, washed 3 times with PBS buffer (pH=7.4) to remove the planktonic bacteria, and 100 μL of DiPGLa-H diluted in a concentration gradient was added. DiPGLa-H was added and incubated in a constant temperature incubator at 37℃ for 24h (the concentration of DiPGLa-H was 0-64μM), while a blank control was set up with only double-distilled water.

M**embrane permeabilization.** Outer membrane integrity assay. The fluorescent probe NPN (Sigma-Aldrich, catalog no. 104043, ≥98%) was used to assess the outer membrane integrity of DiPGLa-H or mucin-treated *E. coli* K88. Briefly, log-medium *E. coli* K88 cells (OD600 = 0.2) were incubated with NPN (10 μM) in 5 mM HEPES buffer (pH 7.4, containing 5 mM glucose) for 30 min, and background fluorescence was recorded for subtraction. (excitation λ = 350 nm, emission λ = 420 nm) using a microplate reader (Varioskan Flash; Thermo, America). Subsequently, 100 μL of cell suspension was mixed with an equal volume of peptide solutions ranging in concentration from 4 to 64 μM in a sterile 96-well black plate. Fluorescence was recorded over time until no further increase in fluorescence was detected. Each test was performed independently, in duplicate, with three biological replicates, and results were converted to percent NPN uptake using the equation. NPN uptake (%) = (F_obs_ – F_buffer_)/ (F_positive_ – F_buffer_) ×100%.

where Fobs is the observed fluorescence at a given peptide concentration, Fbuffer is the fluorescence of the buffer, and Fpositive is the fluorescence of NPN with microbial cells upon addition of 10 μg/mL polymyxin B (Sigma), which was used as a positive control because of its strong outer membrane-permeabilizing property. Untreated cell suspension served as the negative control.

Inner membrane integrity assay. *E. coli* K88 cells were washed and resuspended in 0.01 mol l−1 of PBS (pH 7.4) to obtain an OD600 of 0.5, followed by the addition of 10 nmol l−1 of PI (Thermo Fisher Scientific, catalogue no. P1304MP) in the presence of SLAP-S25 or colistin. After incubation for 30 min, fluorescence was measured with the excitation wavelength at 535 nm and emission wavelength at 615 nm.
